# Genetic alterations of prostate cancer: in localized and metastatic prostate cancer

**DOI:** 10.1186/s12894-025-01840-5

**Published:** 2025-07-14

**Authors:** Chang Eil Yoon, San Kang, Seung ah Rhew, Hyeok Jae Kwon, Dongho Shin, Hyong Woo Moon, Mee Young Kim, Ji Youl Lee

**Affiliations:** 1 Department of Urology, Seoul St. Mary’s Hospital, College of Medicine, The Catholic University of Korea, 222 Banpo-daero, Seocho-gu, Seoul, 06591 Republic of Korea; 2 Catholic Prostate Institute, The Catholic University of Korea, Seoul, Republic of Korea; 3 Cancer Research Institute, College of Medicine, The Catholic University of Korea, Seoul, Republic of Korea

**Keywords:** Genetic mutation, Next-generation sequencing, Prostate cancer

## Abstract

**Purpose:**

The purpose of this study was to identify genetic mutations in patients with localized and metastatic prostate cancer, exploring the relationship between these mutations and clinical outcomes.

**Materials and methods:**

We conducted next-generation sequencing on tissue samples from 106 prostate cancer patients at Seoul St. Mary’s Hospital, analyzing prostate-specific antigen (PSA) levels, tumor, node, metastasis (TNM) staging, Gleason score (GS), and clinical course, including treatment modalities and biochemical recurrence (BCR).

**Results:**

The study included 65 patients with localized and 41 with metastatic prostate cancer. Significant differences were noted in PSA levels, T stage, GS, and treatment modalities. We observed prevalent single-nucleotide variations (SNVs), copy number variations (CNVs), and structural variations including gene fusions like TMPRSS2-ERG. Key predictors of metastatic prostate cancer identified were T stage, GS, PIK3CA, LRP6, LRRK2, and APOBEC3B deletion. BRCA2, BCL6, and CHEK2 were significant predictors for BCR.

**Conclusion:**

Our findings suggest that specific genetic mutations, including PIK3CA, LRP6, LRRK2, and BRCA2, are linked to prostate cancer metastasis and BCR. These results highlight the potential of genetic analysis in forecasting the prognosis of prostate cancer patients and guiding therapeutic decisions.

**Supplementary Information:**

The online version contains supplementary material available at 10.1186/s12894-025-01840-5.

## Introduction

Prostate cancer is one of the most commonly diagnosed cancers in males, and its prevalence is increasing. In South Korea, there were 16,815 new cases of prostate cancer in 2020, ranking it as the sixth most common cancer overall and the third most common male cancer, following lung and stomach cancer. Recent global burden assessments revealed that the incidence and mortality of prostate cancer have been rising steadily, with substantial variations across geographic regions and levels of socioeconomic development. As reported in a comprehensive analysis based on the Global Burden of Disease (GBD) Study, both the age-standardized incidence rate and the disability-adjusted life years attributable to prostate cancer have increased notably from 1990 to 2021, particularly in countries with a high Socio-demographic Index [1]. Treatment options for prostate cancer vary depending on the cancer stage and include active surveillance, surgery, and systemic treatment, as outlined in the American Urological Association Guidelines [2, 3]. Additionally, emerging local treatment modalities such as focal therapy—including high-intensity focused ultrasound (HIFU), transurethral ultrasound ablation, and focal laser ablation—have shown promise in selected patients with localized prostate cancer, although high-quality evidence from randomized trials is still needed to confirm their long-term efficacy and safety [4].

However, treatment options for prostate cancer are limited due to its heterogeneity, leading to challenges for urologists. Notably, around 20–30% of prostate cancer patients progress to castration-resistant prostate cancer (CRPC) after androgen deprivation therapy (ADT) [5].

As conventional treatments reach their limits and advances in genetic analysis techniques, such as next-generation sequencing (NGS), are made, interest in genetic analysis and targeted therapy has grown [6, 7]. NGS enables a deeper understanding of the prostate cancer genome, revealing mutations in genes like the androgen receptor (*AR*) and DNA repair pathways. Some mutations, such as *BRCA1*/*2*, are associated with unfavorable prognoses [8].

While many studies have focused on the genetic analysis of prostate cancer, a lack of research has investigated the clinical implications and prognosis associated with these genetic mutations, especially in Koreans. Therefore, this study aimed to identify genetic mutations in Korean patients with localized and metastatic prostate cancer and assess their impact on each patient’s clinical outcome.

## Materials and methods

### Patients and NGS samples

We retrospectively analyzed 106 prostate cancer patients treated at Seoul St. Mary’s Hospital, obtaining informed consent for genetic analysis. A total of 54 prostate biopsy samples and 52 surgical excision samples were analyzed by NGS between July 1, 2021, and May 31, 2023.

We evaluated baseline characteristics such as prostate-specific antigen (PSA) scores, TNM staging, Gleason scores (GS), and clinical outcomes including additional treatment and biochemical recurrence (BCR).

Age was measured based on the timing of tissue specimen collection, and PSA was measured from blood samples taken within a maximum of 8 weeks before tissue collection. The TNM stage was based on the American Joint Committee on Cancer (AJCC) Cancer Staging Manual, Eighth Edition (2017), published by Springer International Publishing [9].

Radical prostatectomies were performed by 5 experienced surgeons. Tissue examination was conducted by pathologists in our hospital, and pathologic staging, the GS, and other factors were confirmed through this pathologic report.

Hormonal therapy (HTx) is a treatment that decreases androgen levels or blocks androgen action and is applied in 2 ways: as preoperative neoadjuvant therapy in locally advanced prostate cancer and as primary therapy in metastatic prostate cancer.

Radiation therapy (RTx) is used for 3 primary purposes: as a first-line treatment, for salvage RTx (in cases of biochemically recurrent prostate cancer following radical prostatectomy), and as palliative RTx for metastatic lesions.

BCR refers to an increase in PSA levels after primary definitive therapy. BCR is specifically defined as a PSA level of 0.2 ng/mL or higher after radical prostatectomy or when the PSA value increases by 2 ng/mL or more above the nadir following RTx [10, 11]. BCR time is defined as the number of months from the occurrence of BCR after first-line treatment.

Patients were categorized into localized or metastatic groups to correlate genetic mutations with clinical outcomes. Metastatic cancer was defined as N1 or M1 stage or higher,

### NGS

Fresh frozen tissue and formalin-fixed paraffin embedding samples were collected. Following DNA extraction, Library preparation was conducted using the CancerSCAN Library Preparation kit and CancerSCAN IO+ (GENINUS Inc., Seoul, Republic of Korea), which is a pan-cancer panel that analyzes 407 cancer-related DNA variants. Finally, the Illumina sequencing system, named NextSeq 550Dx (Illumina, Inc., California, USA), was used for NGS.

### Data analysis

For categorical variables, frequencies and proportions were derived using the Chi-squared test. For continuous variables, median and interquartile ranges were provided using the Mann-Whitney U test. Univariate analysis was performed using the logistic regression model to evaluate the predictive value of genes associated with localized or metastatic prostate cancer, along with BCR. A confirmative PSA level of > 0.2 ng/dL was considered BCR. Variables showing a *p*-value of less than 0.05 in univariate analysis were selected for multivariate analysis. Essential variables, such as the GS and T stage, were also included in the multivariate analysis. Study data are presented as the mean ± standard deviation (SD) or proportions for continuous or categorical variables. Data were considered significant at *p-*values of less than 0.05, and statistical analyses were performed using R version 4.3.1.

## Results

### Comparison of characteristics of patients with localized and metastatic prostate cancer

The baseline characteristics of patients with localized and metastatic prostate cancer are summarized in Table 1. The average age was similar between groups at 69.6 for localized and 71.5 for metastatic (*p* = 0.284). PSA levels were notably higher in the metastatic group (27.1 ng/ml for localized, 396.0 ng/ml for metastatic), showing a significant difference (*p* = 0.006).

**Table 1:** Baseline characteristics comparing localized and metastatic prostate cancer

	L (*N* = 65)	M (*N* = 41)	*p*
Age	69.6 ± 8.6	71.5 ± 8.8	0.284
PSA (ng/mL)	27.1 ± 40.9	396.0 ± 808.5	0.006
T stage			< 0.001
T2	22 (33.8%)	1 ( 2.4%)	
T3a	23 (35.4%)	8 (19.5%)	
T3b	15 (23.1%)	14 (34.1%)	
T4	5 ( 7.7%)	18 (43.9%)	
N stage			< 0.001
N0	65 (100.0%)	16 (39.0%)	
N1	0	25 (61.0%)	
M stage			< 0.001
M0	65 (100.0%)	9 (22.0%)	
M1a	0	1 ( 2.4%)	
M1b	0	27 (65.9%)	
M1c	0	4 ( 9.8%)	
Gleason score			< 0.001
G6(3 + 3)	4 ( 6.2%)	0	
G7(3 + 4)	24 (36.9%)	3 ( 7.3%)	
G7(4 + 3)	20 (30.8%)	6 (14.6%)	
G8(4 + 4)	6 ( 9.2%)	8 (19.5%)	
G9(4 + 5)	7 (10.8%)	17 (41.5%)	
G9(5 + 4)	4 ( 6.2%)	4 ( 9.8%)	
G10(5 + 5)	0	3 ( 7.3%)	
Sampling method			
Biopsy	19 (29.2%)	35 (85.4%)	
Prostatectomy	46 (70.8%)	6 (14.6%)	
Sampled organ			
Primary prostate	65 (100%)	38 (92.7%)	
Brain (metastasis)	0	2 (4.9%)	
Liver (metastasis)	0	1 (2.4%)	
Pre-op HTx	19 (29.2%)	39 (95.1%)	< 0.001
Radical prostatectomy	50 (76.9%)	10 (24.4%)	< 0.001
Surgical margin involvement	15/50 (30.0%)	4/10 (40.0%)	0.804
RTx	9 (13.8%)	4 (9.8%)	0.748
First line treatment	1/9 (11.1%)	0	
Salvage RTx	8/9 (88.9%)	1/4 (25%)	
Palliative RTx	0	3/4 (75%)	
BCR	13 (20.0%)	13 (31.7%)	0.257
BCR time (months)	4.2 ± 16.7	3.3 ± 6.5	0.709

PSA, Prostate-Specific antigen; Pre-op HTx, Pre-operative hormonal therapy; RTx, Radiation therapy; BCR, Biochemical recurrence

In the localized group, the T-stage distribution was as follows: 22 patients (33.8%) were classified as T2, 23 patients (35.4%) as T3a, 15 patients (23.1%) as T3b, and 5 patients (7.7%) as T4. In the metastatic group, the T-stage distribution was significantly different. One patient (2.4%) was classified as T2, 8 patients (19.5%) as T3a, 14 patients (34.1%) as T3b, and 18 patients (43.9%) as T4.

For the N stage, 25 patients (61.0%) were classified as N1, signifying regional lymph nodal involvement.

For the M stage, 27 patients (65.9%) were identified as M1b, and 4 patients (9.8%) were identified as M1c, indicating distant metastasis to a location other than lymph nodes or bones.

GS distribution revealed differences (*p* < 0.001): localized groups primarily had G7 (3 + 4) at 36.9% and G7 (4 + 3) at 30.8%, whereas metastatic groups showed a higher prevalence of higher grades like G9 (4 + 5) at 41.5% and G10 (5 + 5) at 7.3%.

In the localized group, prostatectomy was the predominant sampling method with 46 cases (70.8%), while in the metastatic group, biopsies were more common, used in 35 cases (85.4%). All samples from the localized group were from the primary prostate, while in the metastatic group, 38 samples (92.7%) were from the primary prostate, two samples (4.9%) were from brain metastases, and one sample (2.4%) from liver metastasis.

Pre-operative HTx was administered to 29.2% of patients in the localized group and 95.1% in the metastatic group, with a significant difference (*p* < 0.001). Radical prostatectomy was more common in the localized group (76.9%) compared to the metastatic group (24.4%). Surgical margin involvement was similar between the groups, with no significant difference (*p* = 0.804).

RTx was given to 13.8% of localized and 9.8% of metastatic patients. Among those who underwent RTx, 11.1% in the localized group had it as a first-line treatment, while 88.9% had salvage RTx. In the metastatic group, 25% had salvage RTx, and 75% received palliative RTx.

BCR occurrence was observed in 20.0% of the localized group and 31.7% of the metastatic group, with no significant difference (*p* = 0.257). The average time to BCR was 4.2 months with an SD of ± 16.7 in the localized group and 3.3 months with an SD of ± 6.5 in the metastatic group, with no significant difference (*p* = 0.709).

### Distribution of genetic alterations in prostate cancer patients

**Fig. 1:**
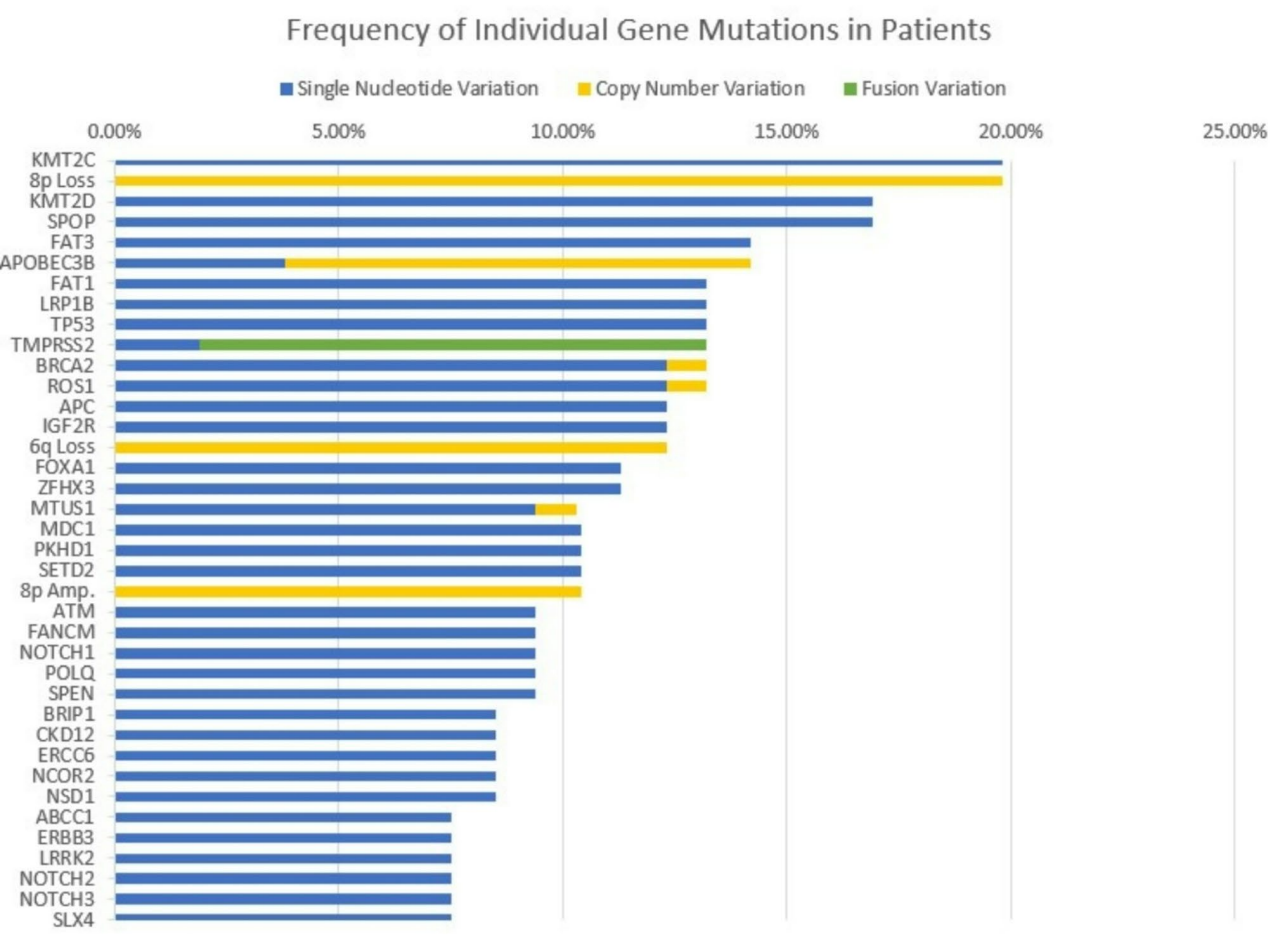
Frequency of Individual Gene Mutations in Patients

**Fig. 2:**
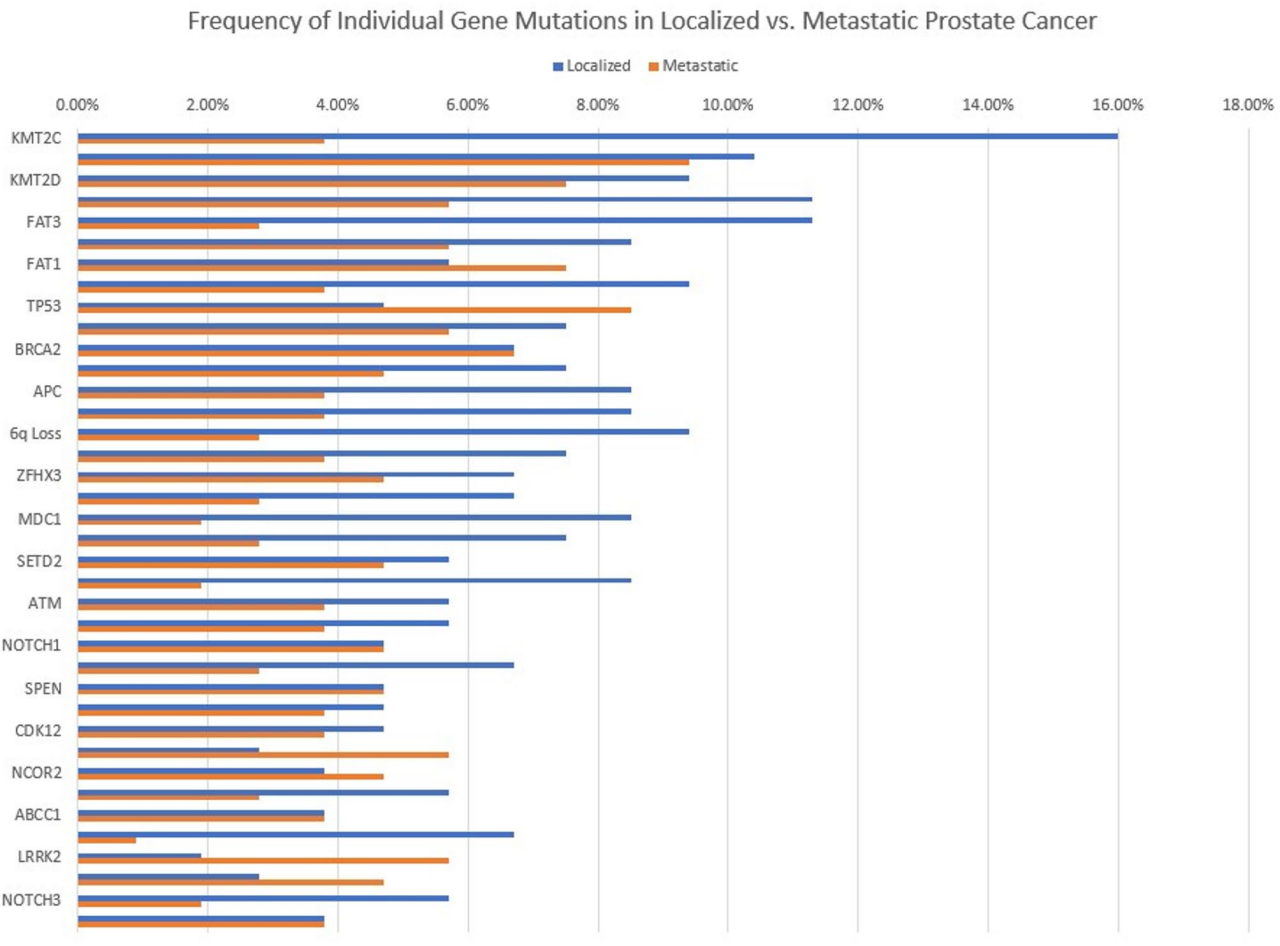
Frequency of Individual Gene Mutations in Localized vs. Metastatic Prostate Cancer

Genetic alterations in these patients included single-nucleotide variations (SNVs), copy number variations (CNVs), and structural variations. A total of 275 SNVs, 60 CNVs, and 17 structural variations were detected. The most common SNVs observed in this study included alterations in genes such as *KMT2C*, *KMT2D*, *SPOP*, *FAT3*, *FAT1*, *LRP1B*, *TP53*, *BRCA2*, *IGF2R*, *ROS1*, *FOXA1*, *ZFHX3*, *MDC1*, *PKHD1*, *SETD2*, *APOBEC3B*, *ATM*, *FANCM*, *MTUS1*, *NOTCH1*, and *POLQ*. These SNVs were found in varying frequencies, with *KMT2C* (19.8%), *KMT2D* (16.9%), and *SPOP* (16.9%) being the most prevalently detected (Fig. 1). To compare mutation profiles between disease stages, we analyzed the frequency of individual gene mutations in localized and metastatic prostate cancer groups (Fig. 2). In terms of CNVs, 8p loss was the most frequent and was detected in 19.8% of patients, followed by 6q loss in 12.3% and *FCGR2B* deletion in 7.5%. Structural variations, including gene fusions, were also noted. *TMPRSS2-ERG* fusion was observed in 8.5% of the patients, while *KMT2C-BAGE2* fusion was found in 2.8% of the cohort (Table 2).

**Table 2:** Distributions of most common genetic alterations

Genetic alterations	Frequency (*N* = 106)	Localized (*N* = 65)	Metastatic (*N* = 41)
Single Nucleotide Variation			
*KMT2C*	21 (19.8%)	17	4
*KMT2D*	18 (16.9%)	10	8
*SPOP*	18 (16.9%)	12	6
*FAT3*	15 (14.2%)	12	3
*FAT1*	14 (13.2%)	6	8
*LRP1B*	14 (13.2%)	10	4
*TP53*	14 (13.2%)	5	9
*APC*	13 (12.3%)	9	4
*BRCA2*	13 (12.3%)	7	6
*IGF2R*	13 (12.3%)	9	4
*ROS1*	13 (12.3%)	8	5
*FOXA1*	12 (11.3%)	8	4
*ZFHX3*	12 (11.3%)	7	5
*MDC1*	11 (10.4%)	9	2
*PKHD1*	11 (10.4%)	8	3
*SETD2*	11 (10.4%)	6	5
*ATM*	10 (9.4%)	6	4
*FANCM*	10 (9.4%)	6	4
*MTUS1*	10 (9.4%)	7	3
*NOTCH1*	10 (9.4%)	5	5
*POLQ*	10 (9.4%)	7	3
*SPEN*	10 (9.4%)	5	5
*BRIP1*	9 (8.5%)	5	4
*CKD12*	9 (8.5%)	5	4
*ERCC6*	9 (8.5%)	3	6
*NCOR2*	9 (8.5%)	4	5
*NSD1*	9 (8.5%)	6	3
Copy number variation			
*8p* Loss	21 (19.8%)	11	10
*6q* Loss	13 (12.3%)	10	3
*FCGR2B* Deletion	8 (7.5%)	5	3
Structural variation			
*TMPRSS2– ERG*	9 (8.5%)	4	5
*KMT2C– BAGE2*	3 (2.8%)	3	0

### Predictive factors for metastatic prostate cancer

Table 3 presents the results of univariate and multivariate analyses assessing the predictive value of various factors in relation to localized and metastatic prostate cancer.

**Table 3:** Univariate and multivariate logistic regression analyses to assess the predictive value of T stage, Gleason score, genetic mutations in relation from localized to metastatic prostate cancer

	Coefficient (95% CI)	*P*
Univariate analysis		
T stage	0.24 (0.17, 0.32)	< 0.001
GS	0.16 (0.11, 0.22)	< 0.001
*KMT2C*	-0.24 (-0.48, -0.01)	0.039
*LRP6*	0.63 (0.07, 1.19)	0.027
*LRRK2*	0.39 (0.04, 0.74)	0.028
*PIK3CA*	0.64 (0.16, 1.12)	0.01
*TP53*	0.3 (0.02, 0.57)	0.035
*APOBEC3B* Amplification	-0.41 (-0.81, -0.01)	0.046
*APOBEC3B* Deletion	0.64 (0.21, 1.07)	0.004
Multivariate analysis		
T stage	0.15 (0.06, 0.23)	0.001
GS	0.06 (0, 0.13)	0.048
*KMT2C*	-0.16 (-0.37, 0.05)	0.133
*LRP6*	0.64 (0.14, 1.14)	0.012
*LRRK2*	0.4 (0.08, 0.72)	0.016
*PIK3CA*	0.55 (0.12, 0.99)	0.013
*TP53*	0.12 (-0.14, 0.37)	0.364
*APOBEC3B* Amplification	-0.3 (-0.67, 0.06)	0.099
*APOBEC3B* Deletion	0.69 (0.3, 1.08)	< 0.001

GS, Gleason score

In the univariate analysis, several factors, namely T stage, GS, *LRP6*, and *LRRK2*, demonstrated strong associations with metastatic prostate cancer. *PIK3CA*, *TP53*, and *APOBEC3B* deletion also showed significant relationships.

Subsequently, the multivariate analysis, focusing on these previously identified significant factors, confirmed T stage, GS, *LRP6*, *LRRK2*, *PIK3CA*, and *APOBEC3B* deletion as significant predictors of metastatic prostate cancer. However, *KMT2C* (*p* = 0.133) and *TP53* (*p* = 0.364) did not achieve statistical significance in the multivariate analysis.

### Predictive factors for BCR in prostate cancer

Table 4 shows the predictive value of GS and genetic mutations in relation to BCR in prostate cancer.

**Table 4:** Univariate and multivariate logistic regression analyses to assess the predictive value of Gleason score and genetic mutations in relation to biochemical recurrence in prostate cancer

	Coefficient (95% CI)	*P*
Univariate analysis		
GS	0.1 (0.05, 0.15)	< 0.001
*BCL6*	0.77 (0.17, 1.37)	0.012
*BRCA2*	0.33 (0.09, 0.58)	0.008
*CHEK2*	0.78 (0.3, 1.26)	0.002
*CREBBP*	0.77 (0.17, 1.37)	0.012
*EPHB4*	0.77 (0.17, 1.37)	0.012
*ERCC2*	0.77 (0.17, 1.37)	0.012
*MET*	0.52 (0.1, 0.95)	0.017
*NPM1*	0.77 (0.17, 1.37)	0.012
*PDGFRB*	0.78 (0.3, 1.26)	0.002
*PLCG1*	0.77 (0.17, 1.37)	0.012
*SMO*	0.77 (0.17, 1.37)	0.012
Multivariate analysis		
GS	0.05 (0.01, 0.09)	0.024
*BCL6*	0.92 (0.48, 1.36)	< 0.001
*BRCA2*	0.36 (0.17, 0.55)	< 0.001
*CHEK2*	0.59 (0.13, 1.04)	0.012
*CREBBP*	1.01 (0.47, 1.55)	< 0.001
*EPHB4*	0.07 (-0.6, 0.74)	0.83
*ERCC2*	-0.18 (-0.8, 0.44)	0.56
*MET*	0.34 (-0.02, 0.7)	0.062
*NPM1*	0.83 (0.3, 1.36)	0.003
*PDGFRB*	0.73 (0.28, 1.18)	0.002
*PLCG1*	0.23 (-0.43, 0.88)	0.495
*SMO*	0.88 (0.33, 1.43)	0.002

GS, Gleason score

In univariate analysis, GS demonstrated a strong predictive value (coefficient = 0.1, 95% confidence interval (CI): 0.05 to 0.15, *p* < 0.001) for BCR. Additionally, genetic mutations, including *BRCA2*, *BCL6*, *CHEK2*, and others, were also considered valuable predictors of BCR in prostate cancer.

The multivariate analysis confirmed the predictive value of GS for BCR. Several genetic mutations, including *BRCA2*, *BCL6*, *CHEK2*, *CREBBP*, *NPM1*, and others, continued to exhibit substantial predictive significance.

## Discussion

In this study, PSA levels were measured in the majority of patients shortly before tissue collection. However, for some patients, this was not feasible and resulted in a maximum time gap of up to 6 weeks. Parameters related to PSA levels, including PSA velocity and PSA doubling time, are frequently mentioned as factors for predicting disease progression [12]. In cases of advanced and aggressive prostate cancer, PSA velocity is notably high, so a 6-week interval might have exhibited a sufficient difference in PSA levels. It is believed that future well-controlled studies could yield even more significant results.

A total of 13 cases of BCR were identified in the group of localized prostate cancer patients. Among them, 10 patients underwent radical prostatectomy, 2 patients received HTx, and 1 patient underwent neoadjuvant HTx, followed by radical prostatectomy. Notably, among the 10 patients who had a radical prostatectomy, surgical margin involvement was confirmed in 7 cases, and BCR occurred relatively shortly after the procedure (ranging from 2 months to 16 months).

The role of surgical margin involvement as a predictor of BCR was established in previous studies [13–15]. Considering this, bias may arise when assessing the impact of genetic mutations on BCR. However, according to a document published in 2014, BCR is correlated not only with surgical margin involvement but also with factors such as the pathologic stage, GS, preoperative PSA level, and tumor volume [16].

In our study, among the 7 patients with confirmed surgical margin involvement, only one was pathologically staged as T2, while the rest were all at T3 or a higher stage. Additionally, when considering factors like GS and PSA levels, these patients are categorized as “unfavorable intermediate” or a higher risk group according to the National Comprehensive Cancer Network (NCCN) guidelines.

To sum up these findings, it is important to exercise caution in result interpretation, as multiple factors contribute to progressive disease.

The NCCN guidelines for prostate cancer recommend genetic testing for patients with prostate cancer if they meet specific criteria outlined in the guidelines. These criteria include having a family history of high-risk germline genetic mutation, a diagnosis of metastatic prostate cancer, or classification into a high-risk group (version 2.2022). The guidelines suggest testing for particular genes associated with homologous recombination repair, such as *BRCA1*, *BRCA2*, *ATM*, *PALB2*, and *CHEK2*, as well as genes related to unfavorable genomics, including defects in *PTEN*, *TP53*, and *RB1*. In our study, these genes were also observed in several cases, with *BRCA2*, *ATM*, and *TP53* being the most prominent (12.3%, 9.4%, and 13.2%, respectively), while other genes were detected in less than 5% of cases.

Several studies found that DNA damage repair (DDR) gene mutations were associated with adverse prognoses. Numerous studies revealed that *BRCA2* mutation, which has been closely associated with breast and ovarian cancers, was linked to poor prognosis in patients with prostate cancer [17]. *BRCA2* mutations contribute to a 2–6 fold relative risk for prostate cancer and have been identified in approximately 5–13% of metastatic prostate cancer patients [18]. A retrospective study found that germline and/or somatic DDR defects were identified in 10% of primary tumors and 27% of metastatic samples [19]. Slightly different, another study revealed that *AR*, *TP53*, and *RB1* mutations were more prevalent in metastatic cancers compared to primary tumors. However, DDR mutations showed a similar frequency in both primary and metastatic cancers [20].

In our study, a total of 13 cases with *BRCA2* mutations were identified, with 7 cases (10.8%) in the localized group and 6 cases (14.6%) in the metastatic group. Univariate and multivariate analyses confirmed their statistical significance in relation to BCR, even though an association with metastatic carcinoma was not found.

The ataxia telangiectasia mutated (*ATM*) gene is known as a DNA repair gene. *ATM* plays a role in maintaining genomic integrity and cellular responses to DNA damage. Despite its high frequency, our study did not find any statistically significant results. However, in other studies, *ATM* loss was notably higher in high-grade prostate cancer with a GS of 9 or higher [21]. Additionally, a study involving the Polish population compared patients with mutations in the *BRCA2*, *NBN*, and *ATM* genes and those without these mutations. The findings revealed a significant difference in the incidence of high-grade prostate cancer, with rates of 56% vs. 21% and an odds ratio of 4.7, showing statistically significant results (*p* = 0.0003) [22].

*TP53* mutation was found to be associated with shorter radiographic progression-free survival and more metastases [23]. In our study, 5 cases were found in patients with localized prostate cancer and 9 with metastatic prostate cancer. This was statistically associated with metastatic prostate cancer but confirmed only in univariate analysis.

*AR* mutations are known to be associated with resistance to ADT [24], and a previous study reported that *AR* mutations are detected in 10–30% of prostate cancer patients who progress to CRPC after ADT [25]. In our study, a total of 7 CRPC cases were examined, and 1 *AR* mutation was identified, yielding results similar to the previously mentioned rate (1/7, 14.2%).

We also found several genes with statistically significant but low frequencies, such as *PIK3CA*, *LRRK2*, and *LRP6*. Research on the PI3K pathway is actively progressing worldwide, and its hyperactivation is well known to be associated with prostate cancer initiation and progression [26]. This pathway consists of various subunits (e.g., *PIK3R1*, *PIK3R2*, *PIK3R3*, *PIK3CA*, *PIK3CB*, *PIK3CD*), and *PTEN* mutation is also known to contribute to this pathway. In our study, *PIK3CA* mutations were identified in 3.7%, all within the metastatic group, consistent with proportions of approximately 3–4% reported in other studies [26, 27].

Our study identified mutations in *LRRK2* and *LRP6* in 7.5% and 2.8% of all prostate cancer patients, respectively, with the majority observed in cases of metastatic prostate cancer. However, limited in-depth research has been conducted on these genes. Some papers suggest an association between *LRRK2* and hormone-related cancers, such as breast and prostate cancer [28, 29]. The *LRP6* gene is known to activate the Wnt/β-catenin signaling pathway, which plays a crucial role in cancer progression, demonstrating significant associations with metastatic and recurrent prostate cancer [30]. Thus, additional evidence through further research is necessary in the future.

Although this study did not include pathway-level analyses or functional validation, it provides a macroscopic overview of gene mutation patterns in localized and metastatic prostate cancer. These findings may serve as a foundational dataset for future mechanistic studies aimed at elucidating key oncogenic pathways. Further experimental investigations, including functional assays and single-cell analyses, are warranted to explore the biological significance and pathway involvement of the identified mutations.

There were several limitations to this study. Differences in the genetic analysis results between specimens obtained through a prostate biopsy and those obtained through radical prostatectomy cannot be completely ruled out. Furthermore, while this study only examined somatic mutations from cancer cells, several guidelines emphasize the need to investigate germline mutations [31]. Therefore, it is possible that the results may be different when reanalyzing germline mutations.

In this study, most tumor samples were obtained from the primary prostate, but a small number of metastatic samples were derived from distant metastatic sites, including the brain (*n* = 2) and liver (*n* = 1). While our intention was to capture the genomic landscape of both localized and metastatic prostate cancer, we acknowledge that the inclusion of samples from heterogeneous metastatic sites may introduce biological variability and potentially confound the comparison of mutational profiles. Given the distinct microenvironments and selective pressures of metastatic organs, genetic alterations observed in these tissues may not be directly comparable to those found in primary tumors. To ensure greater biological consistency, future studies should consider limiting genomic analyses to samples derived exclusively from the primary prostate or perform separate subgroup analyses based on the site of tissue origin.

Several previous studies have shown that hormonal therapy can significantly affect the genomic landscape of prostate cancer. In our cohort, only a small number of patients (2 in the localized group and 4 in the metastatic group) underwent prostate biopsy after initiating hormonal therapy, while the remaining 100 patients had biopsy samples obtained prior to any systemic treatment. Although the number of treatment-exposed samples was relatively small and unlikely to have influenced the overall results, we acknowledge that including these samples may introduce potential bias in interpreting the genomic alterations, particularly when distinguishing treatment-induced changes from those associated with natural disease progression.

There were difficulties in conducting statistical analysis in certain subgroups due to the small number of patients. For example, due to the limited availability of metastatic CRPC samples, fewer patients were included in the metastatic CRPC group compared to the non-metastatic hormone-sensitive prostate cancer (HSPC) and metastatic HSPC groups. Additionally, mutations in genes related to the PI3K pathway, including *PIK3R1*, *PIK3CB*, *PIK3CD*, and *PTEN*, were identified, but each was observed in 3 or fewer cases. Enrolling more patients in future studies could allow for a more detailed analysis by subdividing the groups according to cancer stage.

Although several gene mutations showed statistical significance in our analysis, we acknowledge that statistical significance does not always equate to biological or clinical relevance, particularly in studies with relatively small sample sizes. Therefore, further large-scale studies with functional validation are warranted to confirm the clinical implications of these findings.

The follow-up periods for disease progression varied among individuals and were relatively short in some cases. Therefore, new BCRs or the emergence of new gene mutations may occur in the future. Continuous follow-up for BCR represents a challenge for future research.

As this was a retrospective analysis, there were limitations to the results. Conducting a prospective study or performing statistical analyses related to factors such as genetic mutations and overall survival could be helpful in understanding the prognosis of patients with prostate cancer.

A 2021 Lancet article on prostate cancer focused on the advancement of diagnosis and treatment, particularly addressing advancements in genomics and the biology of prostate cancer, as well as improved diagnostic and staging accuracy through the emergence of more sensitive imaging methods [32]. Compared to our study, while some genetic mutations showed differences in prevalence and clinical significance, major mutations like *BRCA*, *TP53*, and *TMPRSS2-ERG* fusion exhibited similarities.

Based on current clinical guidelines, mutations in DDR-related genes such as BRCA2 and CHEK2 are considered indicators for PARP inhibitor therapy, underscoring their clinical relevance. Similarly, alterations in Wnt/β-catenin pathway may represent emerging targets for pathway-specific treatment. These insights support the potential application of genomic data for risk stratification and individualized therapeutic strategies in prostate cancer management.

To our knowledge, this was the first study to compare localized and metastatic prostate cancer in Koreans using NGS. Thus, this study could expand the genetic landscape not only in Koreans but also in populations worldwide and significantly contribute to advancing clinical management, such as personalized therapy.

## Conclusion

The genetic analysis of prostate cancer enabled the analysis of numerous gene mutations and the identification of clinically significant mutations. Several gene mutations, including *PIK3CA*, *LRP6*, *LRRK2*, and *BRCA2*, were found to be associated with metastatic prostate cancer and BCR.

The prognosis of patients with prostate cancer can be predicted by genetic analysis, and a better prognosis can be expected by applying proper targeted therapy for each pathway alteration.

## Electronic supplementary material

Below is the link to the electronic supplementary material.

Supplementary Material 1

## Data Availability

All data generated or analysed during this study are included in this published article and its supplementary information files.
