# Supplementary material for: Genetic alterations of prostate cancer: in localized and metastatic prostate cancer
Source: BMC Urol. 2025 Jul 14;25:166. doi: 10.1186/s12894-025-01840-5 (PMC12257693; doi:10.1186/s12894-025-01840-5)
Supplement: Supplementary file 1 — Supplementary Material 1 [file 12894_2025_1840_MOESM1_ESM.docx]

Supplementary Table. Details of Samples of Tumor Tissue for Localized and Metastatic Prostate

Cancer Patients.

| Localized | Sampling method | Sampled organ | Proportion of tumor tissue (%) | Treatment  Naïve vs Exposed |
| --- | --- | --- | --- | --- |
| L-1 | Prostatectomy | Primary prostate | 50 | Naïve |
| L-2 | Prostatectomy | Primary prostate | 27.27 | Naïve |
| L-3 | Prostatectomy | Primary prostate | 2.96 | Naïve |
| L-4 | Prostatectomy | Primary prostate | 70 | Naïve |
| L-5 | Prostatectomy | Primary prostate | 14.81 | Naïve |
| L-6 | Prostatectomy | Primary prostate | 24.16 | Naïve |
| L-7 | Prostatectomy | Primary prostate | 7.6 | Naïve |
| L-8 | Prostatectomy | Primary prostate | 11.4 | Naïve |
| L-9 | Prostatectomy | Primary prostate | <1 | Naïve |
| L-10 | Prostatectomy | Primary prostate | 3.04 | Naïve |
| L-11 | Prostatectomy | Primary prostate | 8.8 | Naïve |
| L-12 | Prostatectomy | Primary prostate | 30.51 | Naïve |
| L-13 | Prostatectomy | Primary prostate | 4 | Naïve |
| L-14 | Prostatectomy | Primary prostate | 71.79 | Naïve |
| L-15 | Prostatectomy | Primary prostate | 21.43 | Naïve |
| L-16 | Prostatectomy | Primary prostate | 4.85 | Naïve |
| L-17 | Prostatectomy | Primary prostate | 30.51 | Naïve |
| L-18 | Prostatectomy | Primary prostate | 2.96 | Naïve |
| L-19 | Prostatectomy | Primary prostate | 4.85 | Naïve |
| L-20 | Biopsy | Primary prostate | 7.22 | Naïve |
| L-21 | Biopsy | Primary prostate | 55.07 | Naïve |
| L-22 | Prostatectomy | Primary prostate | 1.5 | Naïve |
| L-23 | Prostatectomy | Primary prostate | 80 | Exposed |
| L-24 | Biopsy | Primary prostate | 84 | Naïve |
| L-25 | Prostatectomy | Primary prostate | 8 | Naïve |
| L-26 | Biopsy | Primary prostate | 70 | Naïve |
| L-27 | Prostatectomy | Primary prostate | 6.9 | Naïve |
| L-28 | Prostatectomy | Primary prostate | 38.3 | Naïve |
| L-29 | Prostatectomy | Primary prostate | 7.13 | Naïve |
| L-30 | Prostatectomy | Primary prostate | 11 | Naïve |
| L-31 | Prostatectomy | Primary prostate | 2.3 | Naïve |
| L-32 | Prostatectomy | Primary prostate | 4.85 | Naïve |
| L-33 | Prostatectomy | Primary prostate | 18.18 | Naïve |
| L-34 | Prostatectomy | Primary prostate | 46.15 | Naïve |
| L-35 | Prostatectomy | Primary prostate | 17.39 | Naïve |
| L-36 | Prostatectomy | Primary prostate | 48.48 | Naïve |
| L-37 | Biopsy | Primary prostate | 70 | Naïve |
| L-38 | Prostatectomy | Primary prostate | 70 | Naïve |
| L-39 | Prostatectomy | Primary prostate | 75 | Naïve |
| L-40 | Prostatectomy | Primary prostate | 80 | Naïve |
| L-41 | Prostatectomy | Primary prostate | 4.75 | Naïve |
| L-42 | Prostatectomy | Primary prostate | 8.3 | Naïve |
| L-43 | Prostatectomy | Primary prostate | 22 | Naïve |
| L-44 | Prostatectomy | Primary prostate | 5.17 | Naïve |
| L-45 | Prostatectomy | Primary prostate | 43.9 | Naïve |
| L-46 | Biopsy | Primary prostate | 71.03 | Naïve |
| L-47 | Prostatectomy | Primary prostate | 70 | Naïve |
| L-48 | Biopsy | Primary prostate | 70.27 | Naïve |
| L-49 | Biopsy | Primary prostate | 80 | Naïve |
| L-50 | Biopsy | Primary prostate | 80 | Naïve |
| L-51 | Biopsy | Primary prostate | 40 | Naïve |
| L-52 | Prostatectomy | Primary prostate | 80 | Naïve |
| L-53 | Biopsy | Primary prostate | 50 | Naïve |
| L-54 | Prostatectomy | Primary prostate | 50 | Naïve |
| L-55 | Biopsy | Primary prostate | 80 | Naïve |
| L-56 | Biopsy | Primary prostate | 75 | Naïve |
| L-57 | Biopsy | Primary prostate | 80 | Naïve |
| L-58 | Prostatectomy | Primary prostate | 80 | Naïve |
| L-59 | Prostatectomy | Primary prostate | 70 | Naïve |
| L-60 | Biopsy | Primary prostate | 90 | Naïve |
| L-61 | Biopsy | Primary prostate | 70 | Naïve |
| L-62 | Biopsy | Primary prostate | 60 | Naïve |
| L-63 | Biopsy | Primary prostate | 60 | Naïve |
| L-64 | Prostatectomy | Primary prostate | 40 | Exposed |
| L-65 | Biopsy | Primary prostate | 80 | Naïve |
| Metastatic | Sampling method | Organ | Proportion of tumor tissue (%) | Treatment  Naïve vs Exposed |
| M-1 | Prostatectomy | Primary prostate | 80 | Naïve |
| M-2 | Biopsy | Primary prostate | 80 | Naïve |
| M-3 | Biopsy | Primary prostate | 30.51 | Naïve |
| M-4 | Biopsy | Primary prostate | 80 | Naïve |
| M-5 | Biopsy | Primary prostate | 57.14 | Naïve |
| M-6 | Prostatectomy | Primary prostate | 4.85 | Exposed |
| M-7 | Biopsy | Primary prostate | 70 | Naïve |
| M-8 | Biopsy | Primary prostate | 41.27 | Naïve |
| M-9 | Biopsy | Primary prostate | 38.71 | Naïve |
| M-10 | Biopsy | Primary prostate | 90 | Naïve |
| M-11 | Biopsy | Primary prostate | 90 | Naïve |
| M-12 | Biopsy | Brain (metastasis) | 80 | Naïve |
| M-13 | Biopsy | Primary prostate | 12.04 | Naïve |
| M-14 | Biopsy | Primary prostate | 12.54 | Naïve |
| M-15 | Biopsy | Primary prostate | 43.9 | Naïve |
| M-16 | Biopsy | Primary prostate | 75 | Naïve |
| M-17 | Biopsy | Primary prostate | 52.94 | Naïve |
| M-18 | Biopsy | Primary prostate | 68.42 | Naïve |
| M-19 | Prostatectomy | Primary prostate | 4.59 | Exposed |
| M-20 | Biopsy | Primary prostate | 50.75 | Naïve |
| M-21 | Biopsy | Primary prostate | 80 | Naïve |
| M-22 | Biopsy | Brain (metastasis) | 80 | Naïve |
| M-23 | Biopsy | Primary prostate | 70 | Naïve |
| M-24 | Biopsy | Primary prostate | 80 | Naïve |
| M-25 | Biopsy | Liver (metastasis) | 90 | Naïve |
| M-26 | Biopsy | Primary prostate | 80 | Naïve |
| M-27 | Biopsy | Primary prostate | 55 | Naïve |
| M-28 | Biopsy | Primary prostate | 90 | Naïve |
| M-29 | Prostatectomy | Primary prostate | 50 | Exposed |
| M-30 | Biopsy | Primary prostate | 20 | Naïve |
| M-31 | Biopsy | Primary prostate | 80 | Naïve |
| M-32 | Biopsy | Primary prostate | 80 | Naïve |
| M-33 | Biopsy | Primary prostate | 80 | Naïve |
| M-34 | Biopsy | Primary prostate | 70 | Naïve |
| M-35 | Biopsy | Primary prostate | 80 | Naïve |
| M-36 | Biopsy | Primary prostate | 60 | Naïve |
| M-37 | Biopsy | Primary prostate | 90 | Naïve |
| M-38 | Biopsy | Primary prostate | 90 | Naïve |
| M-39 | Prostatectomy | Primary prostate | 80 | Naïve |
| M-40 | Biopsy | Primary prostate | 40 | Naïve |
| M-41 | Prostatectomy | Primary prostate | 60 | Exposed |
